# Employment of Iodide of Potassium, in the Treatment of Aneurisms

**Published:** 1860-06

**Authors:** 


*Employment of Iodide of Potassium in the Treatment of Aneurisms.*—The Gazette des Hospitaux has lately reported a clinical lecture of Professor Bouillaud upon aneurisms, and upon the results of the treatment of aneurismal tumors by the iodide of potassium. One of the patients was a man suffering under an aneurism of the brachio-cephalic trunk and aorta, and the other was a woman with an aneurism of the carotid artery. In the latter case, the iodide of potassium was administered for some days in the dose of a gramme, and afterwards in doses of two grammes for two months. At the end of this period, the tumor, which was at first as large as a pigeon's egg, had diminished so much that it might be considered to have disappeared completely. In the case of the man, the tumor, which was of considerable size, underwent displacement contemporaneously with a very well-marked diminution in value, under the same treatment as that adopted for the woman. At the time of the report, however, the man was still under the treatment by iodide of potassium, and therefore no positive conclusions could be drawn. M. Bouillaud has treated other cases with iodide of potassium. In the case of a man with a large aneurismal tumor at the point of origin of the carotid and subclavian arteries, he found that the swelling was considerably diminished in a few weeks by the use of the iodide. In another patient, treated in the same manner for an aneurism of the carotid artery, he observed at the end of a few weeks that the tumor had almost entirely disappeared. These cases are considered to be sufficiently satisfactory to encourage practitioners in making further trials of the iodide of potassium in aneurismal tumors.—*B. and F. Med.-Chirurg. Rev.*

